# Hypoxia‐Induced Histone Lactylation Promotes Ferroptosis in Cardiomyocytes via the Wnt/β‐Catenin Pathway

**DOI:** 10.1002/kjm2.70123

**Published:** 2025-11-13

**Authors:** Xin‐Hua Zhu, Jing‐Wen Hou, Zhong‐Ying Lv, Ming‐Hui Sun, Xiao‐Yang Zhang, Lei Zhang, Mei Wang

**Affiliations:** ^1^ Department of Geriatrics The Fifth Affiliated Hospital of Xinjiang Medical University Urumqi City China; ^2^ Department of Hypertension The Fifth Affiliated Hospital of Xinjiang Medical University Urumqi City China; ^3^ Department of Nephrology The Fifth Affiliated Hospital of Xinjiang Medical University Urumqi City China

**Keywords:** cardiomyocytes, ferroptosis, glycolysis, histone lactylation, hypoxia

## Abstract

This study investigated the effects of histone lactylation on ferroptosis in hypoxia‐induced cardiomyocytes. A hypoxia model was established in AC16 cells treated with 2‐Deoxy‐D‐glucose (2‐DG, a glucose analogue), ferrostatin‐1 (Fer‐1, a selective ferroptosis inhibitor), lactate (LA), sh‐β‐catenin (shRNA of β‐catenin), or SKL2001 (an agonist of the Wnt/β‐catenin pathway) for subsequent experiments. Hypoxia increased glycolysis ability, HK2, PDK1, and LDHA mRNA expression, HK and LDH activities, and LA levels in AC16 cells, decreased SLC7A11 and GPX4 protein levels and GSH levels, and elevated iron ion, MDA, and ROS levels, Wnt3 and nuclear β‐catenin protein levels, β‐catenin nucleus entry, the overall level of lactylation, the lactylation of β‐catenin, and β‐catenin protein stability. 2‐DG or Fer‐1 treatment reduced iron ion, MDA, and ROS levels but increased SLC7A11 and GPX4 protein levels and GSH levels in hypoxia‐treated cells. 2‐DG treatment decreased Wnt3 and nuclear β‐catenin protein levels, β‐catenin nucleus entry, the overall level of lactylation, the lactylation of β‐catenin, and β‐catenin protein stability, whereas LA treatment produced the opposite effects. Wnt/β‐catenin pathway repression attenuated hypoxia‐induced ferroptosis in cardiomyocytes. Collectively, hypoxia enhances histone lactylation, activates the Wnt/β‐catenin pathway, and increases β‐catenin stability, thereby promoting ferroptosis in cardiomyocytes.

## 1 Introduction

Myocardial infarction (MI) represents a life‐threatening coronary‐associated pathology characterized by sudden cardiac death [1]. MI occurs due to plaque formation in arterial walls, leading to reduced blood flow to the heart and myocardial injury from oxygen deprivation [2]. Ferroptosis, an iron‐dependent form of cell death, plays a critical role in disorders such as cancer and ischemic organ injury [3]. It represents a pivotal type of cardiomyocyte death in infarcted regions and contributes to myocardial pathology in heart disease [4]. Hypoxia induces ferroptosis in cardiomyocytes [5]. Thus, elucidating its mechanisms may improve the management of MI.

Oxygen is essential for cardiomyocyte metabolism, and hypoxia triggers various metabolic changes in hearts [6]. The metabolic changes of cardiomyocytes encompass reduced mitochondrial pyruvate oxidation and elevated lactate (LA) export [7]. LA is a vital product of glycolysis [8], which is frequently altered during cardiovascular diseases [9]. A prior study displayed that LA was up‐regulated in hypoxia‐treated cardiomyocytes [10]. Histone lactylation, a marker of LA levels and glycolysis, regulates cell metabolism and can affect cell fate, including carcinogenesis [11]. Previous research unveiled that histone lactylation participated in modulating immune homeostasis and cardiac function after MI [12]. Hence, it is reasonable to speculate that LA may also play a crucial role in CHF. Reportedly, hypoxia‐induced glycolysis promotes β‐catenin lactylation, enhancing its stability and expression in colorectal cancer cells [13]. Moreover, the Wnt/β‐catenin pathway is tightly implicated in heart development and regeneration [14]. Wnt/β‐catenin pathway activation was observed to attenuate hypoxia/reoxygenation‐stimulated cardiomyocyte injury [15]. Additionally, a previous study demonstrated the promotional impacts of Wnt/β‐catenin pathway activation on neuron ferroptosis in intracerebral hemorrhage [16].

In this context, it is rational to speculate that hypoxia‐induced histone lactylation might drive ferroptosis in cardiomyocytes via the Wnt/β‐catenin pathway. Therefore, this research examined the relationship among LA levels, ferroptosis, and the Wnt/β‐catenin pathway in hypoxia‐induced human AC16 cardiomyocytes.

## 2 Materials and Methods

### 2.1 Cell Culture

Human AC16 cardiomyocytes (CRL‐3568; ATCC, Manassas, Virginia, USA) were maintained in DMEM (Invitrogen, Carlsbad, CA, USA) encompassing 1% antibiotics (100 U/mL penicillin and 100 μg/mL streptomycin; 15,140,122; Invitrogen) and 10% fetal bovine serum (A5670701; Invitrogen) in an incubator (5% CO_2_ and 37°C; Thermo Fisher Scientific, Rockford, IL, USA).

### 2.2 Cell Processing

AC16 cardiomyocytes were cultured in a closed hypoxic incubator (37°C, 5% CO_2_, 1% O_2_, 94% N_2_) for hypoxia induction [17]. Subsequently, AC16 cells were grouped as follows: (a) normoxia group: cells were cultured (24 h) in a normoxic incubator (5% CO_2_, 21% O_2_, and 74% N_2_); (b) hypoxia group: cells were cultured (24 h) in a closed hypoxic incubator (37°C, 5% CO_2_, 1% O_2_, and 94% N_2_); (c) hypoxia +2‐Deoxy‐D‐glucose (2‐DG) group: 10 mM 2‐DG (HY‐13966; MCE, Monmouth Junction, NJ, USA) was added to the culture medium of AC16 cells, followed by 24‐h incubation under hypoxic conditions; (d) hypoxia + Ferrostatin‐1 (Fer‐1) group: 2 μM Fer‐1 (ferroptosis inhibitor; HY‐100579; MCE) was added to the culture medium of AC16 cells, followed by 24‐h culture under hypoxic conditions [18]; (e) hypoxia + LA group: 15 μM LA (HY‐B2227; MCE) was added to the culture medium of AC16 cells, followed by 24‐h incubation under hypoxic conditions [13]; (f) hypoxia + short hairpin RNA (sh)‐negative control (NC) group: AC16 cells underwent transfection with sh‐NC and then 24‐h culture under hypoxic conditions; (g) hypoxia + sh‐β‐catenin group: AC16 cells underwent transfection with sh‐β‐catenin and 24‐h culture under hypoxic conditions; (h) hypoxia + sh‐β‐catenin + SKL group: AC16 cells were transfected with sh‐β‐catenin while 20 μM SKL2001 (S8320; Selleck Chemicals, Houston, TX, USA) was supplemented to their culture medium, followed by 24‐h incubation under hypoxic conditions.

shRNA of β‐catenin (sh‐β‐catenin) and the negative control (sh‐NC) used above were designed and synthesized by Sigma‐Aldrich (St. Louis, MO, USA), and the target sequences of shRNA were: sh‐NC: 5′‐CACCGTTCTCCGAACGTGTCACGTCGAAACGTGACACGTTCGGAGATTTTT‐3′; sh‐β‐catenin: 5′‐GTTGTTATCAGAGGACTAAATATTCAAGAGATATTTAATGTCCTCTGATAACAATTTTT‐3′ [19].

### 2.3 Cell Counting Kit‐8 (CCK‐8) Assay

Cell viability was tested as instructed in the protocols of the CCK‐8 kit (96,992; Sigma‐Aldrich). A microplate reader (Bio‐Rad 680; Bio‐Rad, Hercules, CA, USA) was employed for optical density (OD; 450 nm) detection.

### 2.4 Glycolysis Tests

The ECAR level was assessed using a Glycolysis Stress Test Complete Assay Kit (ab222946; Abcam, Cambridge, UK). The Mitochondrial Stress Test Complete Assay Kit (ab232857; Abcam) was employed for the assessment of the OCR level. Briefly, cells were placed in 96‐well plates and cultured for 1 h in the Seahorse XF DMEM comprising 10 mM glucose, 1 mM sodium pyruvate, and 2 mM l‐glutamine without CO_2_ exposure. Subsequently, each well was injected with oligomycin (final concentration of 1.5 μM), fluorocarbonylcyanophenylhydrazone (final concentration of 1.0 μM), and rotenone/antimycin A (final concentration of 1.0 μM for both), and the data were run and analyzed on an Agilent Seahorse XF analyzer (Agilent, California, USA). All operations strictly followed the protocol of the kits. The experiment was repeated three times independently.

### 2.5 Quantitative Real‐Time Polymerase Chain Reaction (qRT‐PCR)

Total RNA in AC16 cells was isolated using TRIzol reagents (15596026CN; Invitrogen). RNA concentration and purity were determined using a Nanodrop2000 microvolume spectrophotometer (Thermo Fisher Scientific). The value of OD260/OD280 ranged from 1.8 to 2.2, indicating high RNA purity. RNA was reversely transcribed into cDNA using a reverse transcription kit (K1622; Jingke Chemical Technology Co. Ltd., Shanghai, China). Real‐time fluorescence quantitative PCR was carried out with a quantitative PCR instrument (7500; ABI, Foster City, CA, USA). The reaction procedure encompassed 10 min at 95°C and 40 cycles of 10 s at 95°C, 30 s at 60°C, and 30 s at 72°C. The 2^−ΔΔ*Ct*^ method was applied for target gene expression calculation, with GAPDH as the internal reference gene. Primers, which were synthesized by Sangon (Shanghai, China), are listed in Table 1.

**TABLE 1:** Primer sequences for PCR.

Genes	Forward (5′–3′)	Reverse (5′–3′)
HK2	GTGAATCGGAGAGGTCCCAC	CAAGCAGATGCGAGGCAATC
PDK1	AGGCGTTGCAAGTATCACCA	CACACCAAAGCAGGAAAGGC
LDHA	GCCGATTCCGGATCTCATTG	CCAGCCTTTCCCCCATTAGG
Wnt3	ACAACAATGAAGCAGGCCGA	ACTCACGGTGTTTCTCCACC
β‐Catenin	TCTTGCCCTTTGTCCCGCAAATCA	TCCACAAATTGCTGTGTCCCA
GAPDH	GAGTCCACTGGCGTCTTCAC	GTTCACACCCATGACGAACA

### 2.6 Hexokinase (HK) and LA Dehydrogenase (LDH) Activity Determination

After cells were harvested, HK and LDH activities were examined per the protocols of the corresponding kits (ab136957; ab102526; Abcam). In short, after trypsinization, cells were rinsed three times with pre‐cooled phosphate‐buffered saline (PBS), resuspended in pre‐cooled experimental buffer, and centrifuged (12,000 rpm, 5 min, 4°C) for removing insoluble substances. The supernatant was harvested and mixed with the reaction mixture, followed by incubation (room temperature, 40 min) in a dark box. The OD value at 450 nm was determined using the microplate reader (Bio‐Rad 680), followed by relative activity calculation.

### 2.7 LA Measurement

The LA determination kit (A019‐2‐1; Jiancheng Bioengineering Institute, Nanjing, Jiangsu, China) was employed for LA determination. Briefly, cells were lysed using ultrasound on ice, and enzyme solutions and color developer were then added. The OD value at 530 nm was measured using the microplate reader (Bio‐Rad 680), and the LA concentration was calculated based on the standard curve.

### 2.8 Iron Ion Detection

After PBS treatment, cells were centrifuged, and the supernatant was attained. Next, the free iron level was examined using an iron ion assay kit (ab83366; Abcam) following the instructions of the manufacturer. In short, ddH_2_O, iron standard solutions, and samples were added to 96‐well plates, followed by the addition of 200 μL of Fe assay buffers and 8.4 μL of ferrous colorimetric solutions to each well. The reaction samples were mixed evenly and incubated (room temperature, 15 min) away from light. Finally, the OD value was measured at 562 nm.

### 2.9 Malondialdehyde (MDA) and Glutathione (GSH) Detection

MDA (S0131S; Beyotime, Shanghai, China) and GSH (S0056; Beyotime) levels were tested with the corresponding kits as per the instructions. The OD value was determined on the microplate reader (Molecular Devices). MDA and GSH standard samples were employed for generating the standard curve.

### 2.10 Reactive Oxygen Species (ROS) Content Detection

The intercellular ROS content was detected with the ROS fluorescent probe DCFH‐DA molecular probe (HY‐D0940; MCE) following the instructions. Cell supernatants were added with 2 mL of 10 μmol/L DHE probes, followed by culture (37°C, 30 min) away from light. DCFH‐DA was converted to highly fluorescent DCFH upon oxidation and exhibited fluorescence in the cytoplasm. A fluorescence microscope (Olympus IX51; Olympus, Tokyo, Japan) was employed for capturing fluorescence images.

### 2.11 Western Blot (WB)

Nuclear and plasma proteins in AC16 cells were extracted using a nuclear protein extraction kit (R0050; Solarbio, Beijing, China), with protein concentrations measured using a bicinchoninic acid kit (P0010; Beyotime). Protein samples were added with an appropriate amount of loading buffer and then heated (5 min) in a boiling water bath for protein denaturation. After that, 20 μg of denatured protein samples were added into the loading wells, subjected to 10% SDS‐PAGE for separation, and then transferred to PVDF membranes. After blocking (1 h) in 5% skim milk, the membranes were incubated (overnight, 4°C) with the primary antibodies: anti‐Wnt3 (ab116222; 1:1000; Abcam; 40 kDa), anti‐β‐catenin (ab32572; 1:1000; Abcam; 86 kDa), anti‐solute carrier family 7 member 11 (SLC7A11; ab307601; 1:1000; Abcam; 55 kDa), anti‐glutathione peroxidase 4 (GPX4; ab125066; 1:1000; Abcam), anti‐pan‐kla (A23004; 1:1000; ABclonal), anti‐GAPDH (ab9485; 1:1000; Abcam), and anti‐Lamin B (ab32535; 1:500; Abcam). Next, horseradish peroxidase‐conjugated IgG secondary antibody (ab6721; 1:1000; Abcam) were added for 2‐h incubation (room temperature). After three PBS washes (room temperature), the membranes were developed using an enhanced chemiluminescence kit (P0018S; Beyotime). Image J (National Institutes of Health, Bethesda, MD, USA) was employed for protein band density analysis, with GAPDH or Lamin B as the internal reference.

Additionally, the stability of β‐catenin was examined. Cells were treated with cycloheximide (CHX) to inhibit protein synthesis, and β‐catenin levels in cells were detected after 0, 2, 4, and 8 h of CHX treatment. The relative expression of each protein was calculated according to the signal intensity of the protein bands. The half‐life curve of β‐catenin protein was plotted.

### 2.12 Immunofluorescence

Cells were placed on cover glasses, washed twice in PBS, and fixed in methanol for 5 min. Subsequent to 3 PBS washes, cells were treated (30 min) with blocking solutions (1% bovine serum albumin in PBS), washed twice in PBS, and incubated (overnight, 4°C) with anti‐β‐catenin (ab32572; 1:250; Abcam). Following three washes in PBS (5 min/time), cells underwent a 2‐h incubation with conjugated‐goat anti‐rabbit IgG‐Cy3 (ab6939; 1:1000; Abcam). DAPI was employed for nucleus staining. A fluorescence microscope (Olympus IX51) was utilized for cell observation and photographing.

### 2.13 Statistical Analysis

GraphPad Prism 9.5 (GraphPad Software Inc., San Diego, CA, USA) was adopted for data analysis and plotting. The Shapiro–Wilk test was utilized for normal distribution analysis. Normally distributed data, which were represented by mean ± standard deviation, were compared among multiple groups using one‐way analysis of variance, with Tukey's post hoc test. *p* < 0.05 represented a statistically significant difference.

## 3 Results

### 3.1 Hypoxia Elevated LA Levels and Disrupted Glucose Metabolism in Cardiomyocytes

**FIGURE 1:**
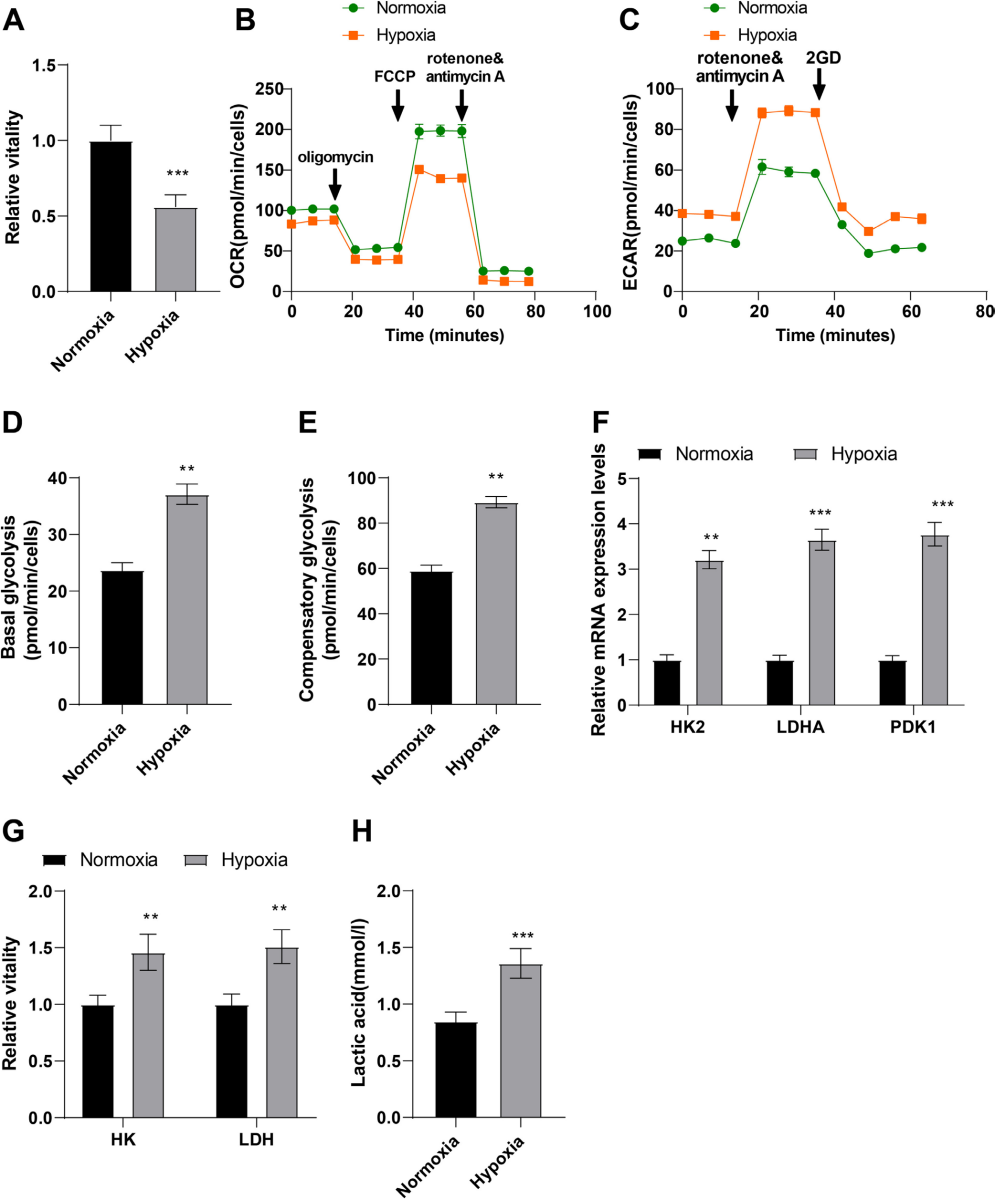
Hypoxia induces elevated LA levels and glucose metabolism disorders in cardiomyocytes. Cardiomyocytes (AC16) were subjected to hypoxia treatment for 24 h. (A) cell viability detected using CCK‐8 assay; (B–E) OCR level, ECAR level, basal glycolysis and glycolysis capacity assessed using kits; (F) the mRNA expression of glycolytic enzymes (HK2, PDK1, and LDHA) in AC16 cells examined using qRT‐PCR; (G) the relative activities of HK and LDH; (H) LA levels. The cell experiment was repeated three times, and the results were expressed as mean ± standard deviation. The independent sample *t*‐test was used for comparisons between the two groups. **p < 0.01, ***p < 0.001.

To ascertain the impact of hypoxia on glucose metabolism in cardiomyocytes, AC16 cells underwent 24‐h hypoxia treatment. As displayed in CCK‐8 assay results, hypoxia significantly reduced AC16 cell viability (*p* < 0.01, Figure 1A). As revealed in Figure 1B,C, hypoxia remarkably reduced the OCR level, elevated the ECAR level, and enhanced AC16 cell basal glycolysis and glycolysis ability (*p* < 0.01). According to qRT‐PCR data, the expression of glycolysis‐related enzymes [hexokinase II (HK2), pyruvate dehydrogenase kinase 1 (PDK1), and LA dehydrogenase A (LDHA)] was dramatically upregulated in hypoxia‐treated cardiomyocytes (all *p* < 0.01, Figure 1D). The relative activities of HK and LDH were also markedly elevated in hypoxia‐treated cardiomyocytes (both *p* < 0.01, Figure 1E). Additionally, LA levels in AC16 cells were noticeably raised after hypoxia induction (*p* < 0.001, Figure 1F). These findings indicated LA upregulation and glucose metabolism disorders in hypoxia‐treated cardiomyocytes.

### 3.2 Histone Lactylation Was Enhanced and Ferroptosis Was Promoted in Hypoxia‐Treated Cardiomyocytes

**FIGURE 2:**
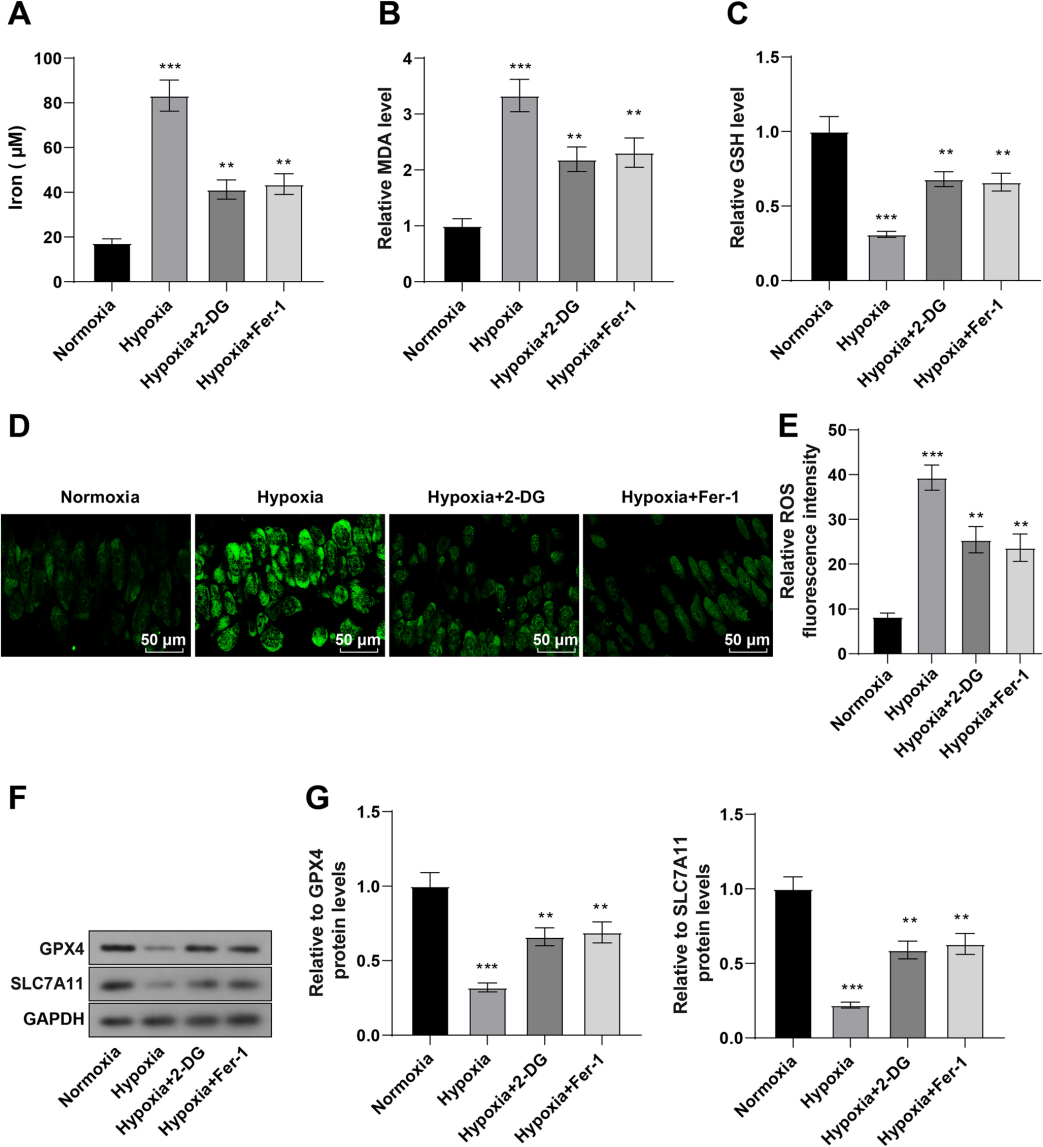
Hypoxia‐treated cardiomyocytes show increased histone lactylation and promoted ferroptosis. AC16 cells were treated with 10 mM 2‐DG or 2 μM Ferrostatin‐1 and incubated in a closed hypoxic incubator (37°C, 5% CO_2_, 1% O_2_, and 94% N_2_) for 24 h. (A) iron ion content; (B, C) levels of lipid peroxidation products MDA (B) and GSH (C) in AC16 cells; (D, E) ROS levels tested with DHE staining; (F, G) SLC7A11 and GPX4 levels detected with WB. The cell experiment was repeated three times, and the results were expressed as mean ± standard deviation. One‐way ANOVA, with Tukey's post hoc test, was used for comparisons among multiple groups. **p < 0.01, ***p < 0.001.

Next, this study analyzed whether histone lactylation affected ferroptosis sensitivity in hypoxia‐treated cardiomyocytes. Fe^2+^, MDA, and GSH levels were first detected, which demonstrated that Fe^2+^ and MDA levels were considerably elevated and GSH levels were reduced in hypoxia‐treated cardiomyocytes (all *p* < 0.001, Figure 2A–C). According to immunofluorescence results, ROS levels were prominently increased in cardiomyocytes after hypoxia induction (all *p* < 0.001, Figure 2D,E). Additionally, the protein levels of ferroptosis‐related proteins (SLC7A11 and GPX4) were lowered in hypoxia‐treated cardiomyocytes (both *p* < 0.001, Figure 2F,G). After 24‐h treatment of 10 mM 2‐DG (a glycolysis inhibitor) or 2 μM Ferrostatin‐1 (a ferroptosis inhibitor), hypoxia‐treated AC16 cells exhibited remarkably decreased Fe^2+^, MDA, and ROS levels, as well as increased GSH, SLC7A11, and GPX4 levels (all *p* < 0.01, Figure 2A–G). Conclusively, hypoxia may promote ferroptosis in cardiomyocytes by enhancing histone lactylation.

### 3.3 Hypoxia‐Induced Histone Lactylation Activated the Wnt/β‐Catenin Pathway and Stabilized β‐Catenin in Cardiomyocytes

**FIGURE 3:**
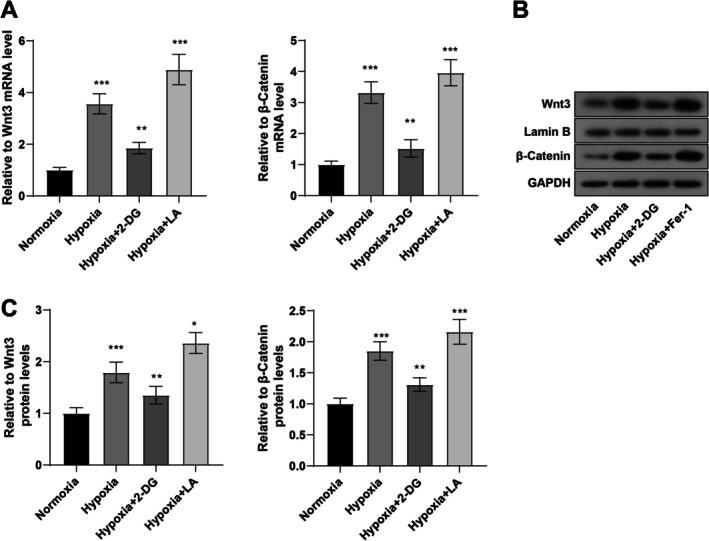
Hypoxia induces activation of the Wnt/β‐catenin pathway in cardiomyocytes. AC16 cells were treated with 10 mM 2‐DG or 15 μM LA and incubated in a closed hypoxic incubator (37°C, 5% CO_2_, 1% O_2_, and 94% N_2_) for 24 h. (A) mRNA expression of Wnt3 and β‐catenin in AC16 cells examined with qRT‐PCR; (B, C) protein level of Wnt3 and nuclear β‐catenin in AC16 cells measured with WB. The cell experiment was repeated three times, and the results were expressed as mean ± standard deviation. One‐way ANOVA, with Tukey's post hoc test, was used for comparisons among multiple groups. *p < 0.05,**p < 0.01, ***p < 0.001.

**FIGURE 4:**
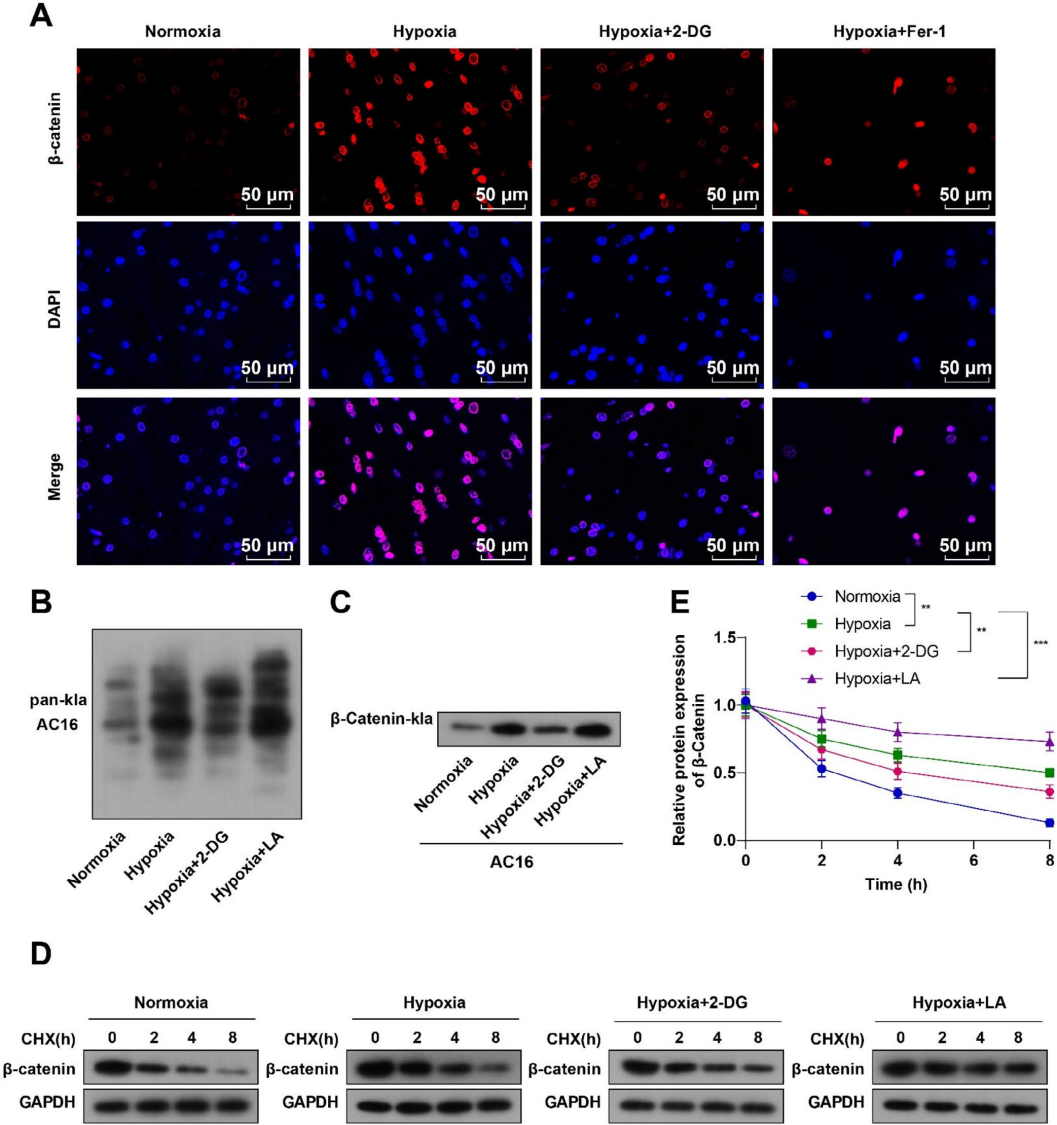
Hypoxia‐induced histone lactylation in cardiomyocytes boosts the Wnt/β‐Catenin pathway activation and enhances β‐catenin stability. AC16 cells were treated with 10 mM 2‐DG or 15 μM LA and incubated in a closed hypoxic incubator (37°C, 5% CO_2_, 1% O_2_, and 94% N_2_) for 24 h. (A) β‐catenin nuclear entry analyzed with immunofluorescence; (B) overall lactylation level in AC16 cells examined with WB; (C) lactylation of β‐catenin in AC16 cells detected with WB; (D, E) AC16 cells were treated with 100 μg/mL CHX for 0, 2, 4, and 8 h, followed by WB detection of β‐catenin protein stability. The cell experiment was repeated three times, and the results were expressed as mean ± standard deviation. One‐way ANOVA, with Tukey's post hoc test, was used for comparison among multiple groups. **p < 0.01, ***p < 0.001.

This study further delved into the mechanism of hypoxia‐caused increased histone lactylation in cardiomyocytes. Wnt3 and β‐catenin mRNA expression was determined with qRT‐PCR, which was strikingly augmented in AC16 cells subsequent to hypoxia induction (both *p* < 0.001, Figure 3A). According to WB results, Wnt3 and nuclear β‐catenin protein levels were conspicuously elevated in AC16 cells under hypoxia conditions (both *p* < 0.001, Figure 3B,C). Furthermore, immunofluorescence data revealed that hypoxia‐treated AC16 cells exhibited increased nuclear entry of β‐catenin (Figure 4A). According to WB results, the overall level of lactylation and the lactylation of β‐catenin in AC16 cells were substantially increased in hypoxia‐induced AC16 cells (Figure 4B,C). Changes in β‐catenin protein stability in AC16 cells after CHX treatment were examined with WB, and increased β‐catenin protein stability in AC16 cells was revealed (*p* < 0.001, Figure 4D,E).

After 2‐DG treatment, hypoxia‐treated AC16 cells showed decreased Wnt3 and nuclear β‐catenin expression and inhibited β‐catenin entry into the nucleus, accompanied by appreciable reductions in the overall level of lactylation, the lactylation of β‐catenin, and β‐catenin protein stability (all *p* < 0.01, Figures 3A–C and 4A–E). LA is an epigenetic regulator that induces lactylation of lysine residues, leading to an increase in the cellular lactylation level [13]. We speculated that LA treatment might produce the opposite phenomenon to cells treated with 2‐DG. LA treatment caused opposite results in hypoxia‐treated AC16 cells, as evidenced by marked increases in Wnt3 and nuclear β‐catenin expression, β‐catenin entry into the nucleus, the overall level of lactylation, the lactylation of β‐catenin, and β‐catenin protein stability (all *p* < 0.001, Figures 3A–C and 4A–E). Taken together, hypoxia‐induced histone lactylation in cardiomyocytes promoted the Wnt/β‐catenin pathway activation and enhanced β‐catenin stability.

### 3.4 Inhibition of the Wnt/β‐Catenin Pathway Attenuated Hypoxia‐Induced Ferroptosis in Cardiomyocytes

**FIGURE 5:**
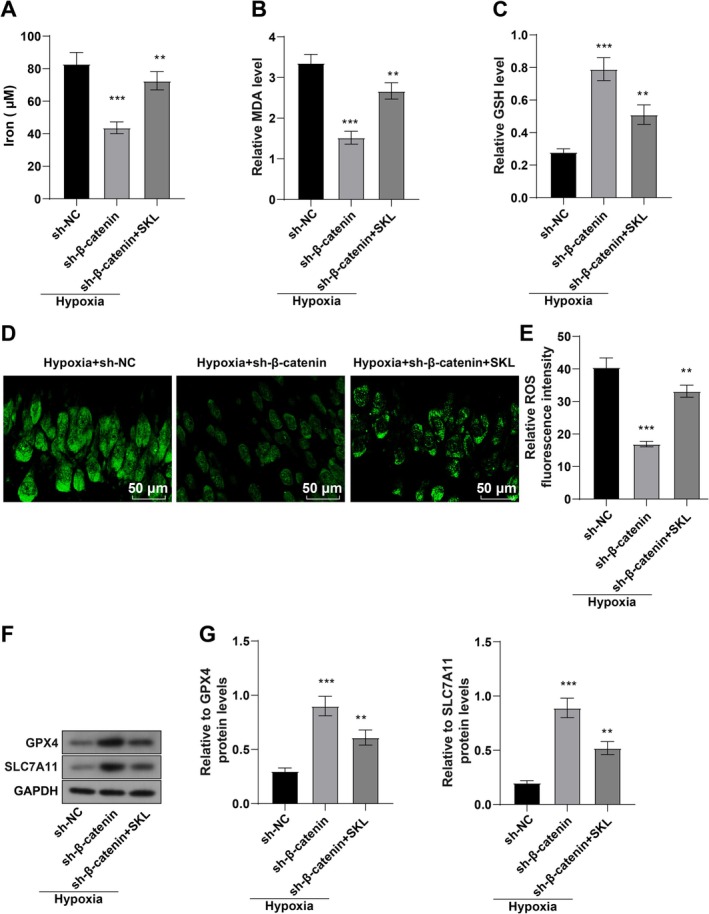
Inhibition of the Wnt/β‐catenin pathway depresses hypoxia‐induced ferroptosis in cardiomyocytes. AC16 cells were transfected with sh‐β‐catenin or treated with 20 μM SKL2001 and incubated in a closed hypoxic incubator (37°C, 5% CO_2_, 1% O_2_, and 94% N_2_) for 24 h. (A) iron ion contents; (B, C) levels of lipid peroxidation products MDA (B) and GSH (C) in AC16 cells; (D, E) ROS levels in AC16 cells determined with DHE staining; (F, G) levels of ferroptosis‐related proteins (SLC7A11 and GPX4) examined with WB. The cell experiment was repeated three times, and the results were expressed as mean ± standard deviation. One‐way ANOVA, with Tukey's post hoc test, was used for comparisons among multiple groups. **p < 0.01, ***p < 0.001.

Finally, this study investigated whether hypoxia promoted ferroptosis in cardiomyocytes by mediating the Wnt/β‐catenin pathway. Briefly, hypoxia‐induced AC16 cells underwent sh‐β‐catenin (β‐catenin‐specific silencing) transfection or 20 μM SKL2001 (an activators of the Wnt/β‐catenin pathway) treatment, to reduce β‐catenin expression or activate the Wnt/β‐catenin pathway. The results indicated that sh‐β‐catenin transfection decreased Fe^2+^, MDA, and ROS levels and increased GSH, SLC7A11, and GPX4 levels in hypoxia‐induced AC16 cells (all *p* < 0.01, Figure 5A–G). Furthermore, 20 μM SKL2001 notably reversed the above results, as evidenced by elevated Fe^2+^, MDA, and ROS levels and reduced GSH, SLC7A11, and GPX4 levels (all *p* < 0.01, Figure 5A–G). Altogether, repression of the Wnt/β‐catenin pathway nullified hypoxia‐triggered ferroptosis in cardiomyocytes.

## 4 Discussion

Hypoxia contributes to cardiovascular diseases [20]. A hypoxic environment is strongly associated with abnormal cellular energy metabolism in various disorders [21]. This study demonstrated that hypoxia‐induced histone lactylation promoted ferroptosis in cardiomyocytes, which involved the activated Wnt/β‐catenin pathway.

It is well established that HK2, LDHA, and PDK1 were effective glycolysis‐related enzymes [22]. Moreover, HK and LDH are potent glucose metabolism‐associated enzymes [23]. It has been shown that glycolytic enzymes (including HK and LDH) play great roles in cardiovascular diseases, such as heart failure and atherosclerosis [24]. Additionally, LA is an end product of LDHA from pyruvate during anaerobic glycolysis [25]. In this study, hypoxia prominently suppressed cardiomyocyte viability and induced glucose dysfunction, as manifested by enhanced cell basal glycolysis and glycolysis ability and upregulated mRNA expression of HK2, PDK1, and LDHA, as well as enhanced relative activities of HK and LDH and increased LA levels. Consistently, a prior study evidenced that glioblastoma cells showed upregulated HK2, LDHA, and PDK1 expression under hypoxia conditions [26]. Another study reported that the activity of HK and LDH was increased to varying degrees under acute hypoxic conditions [27]. Moreover, LA is a byproduct of glycolysis [28]. LA level is a critical health index because of its implications in various diseases, including hypoxia, metabolic disorders, and heart failure [29]. A previous study also demonstrated an increase in the levels of key glycolytic enzymes and LA in the sclera under hypoxia conditions [30]. From the above, it could be concluded that hypoxia‐treated cardiomyocytes showed glucose metabolism disorders and an increase in LA levels.

Histone lactylation is tightly involved in various cellular events, including glycolytic metabolism [31]. Histone lactylation has been shown to induce ferroptosis in alveolar epithelial cells [32]. Iron, MDA, ROS, GSH, SLC7A11, and GPX4 are potent ferroptosis‐associated factors, and ferroptosis is linked to depletion of GSH, SLC7A11, and GPX4, as well as upregulation of iron, MDA, and ROS [33]. The present study explored whether histone lactylation affected ferroptosis in hypoxia‐induced cardiomyocytes. The results revealed that hypoxia strikingly elevated Fe^2+^, MDA, and ROS levels and reduced GSH level in cardiomyocytes, accompanied by downregulated levels of SLC7A11 and GPX4 and that 2‐DG or Ferrostatin‐1 reversed the above trends. Consistently, accumulating studies have provided evidence that hypoxia can provoke ferroptosis, highlighting that ferroptosis promises to be a therapeutic target for hypoxia‐induced diseases [34]. Additionally, a previous study identified that malate dehydrogenase 2 lactylation induced ferroptosis, contributing to cardiovascular diseases (myocardial ischemia–reperfusion injury) [35]. The above findings suggested that hypoxia evoked ferroptosis in cardiomyocytes through enhanced histone lactylation.

Hypoxia can modulate ferroptosis in specific conditions by mediating different pathways [34]. The Wnt/β‐catenin pathway is involved in heart‐related diseases [14]. Furthermore, it has been revealed that alterations in LA production and lactylation levels may affect the Wnt/β‐catenin pathway in periodontal ligament stem cells [36]. Mechanistically, hypoxia‐treated AC16 cells exhibited remarkably upregulated Wnt3 and β‐catenin expression, promoted nuclear entry of β‐catenin, increased overall level of lactylation, augmented lactylation of β‐catenin, and enhanced β‐catenin protein stability. Similarly, prior research revealed that hypoxia treatment remarkably elevated β‐catenin protein levels and lactylation in colorectal cancer cells [13]. This study innovatively demonstrated that 2‐DG treatment diminished Wnt3 and β‐catenin expression, the nuclear entry of β‐catenin, the overall level of lactylation, the lactylation of β‐catenin, and β‐catenin protein stability in hypoxia‐treated AC16 cells, while LA treatment exerted opposite effects, which further validated that hypoxia‐induced histone lactylation promoted Wnt/β‐catenin pathway activation and enhanced β‐catenin stability in cardiomyocytes. Furthermore, the present study disclosed that inhibition of the Wnt/β‐catenin pathway impeded hypoxia‐induced ferroptosis in cardiomyocytes. It has been shown that targeting the Wnt/β‐catenin may be a novel therapeutic method for myocardial infarction [37]. Moreover, a former study unraveled that inactivation of the Wnt/β‐catenin pathway was associated with decreases in ferroptosis‐related protein levels [38].

In summary, this study revealed that hypoxia increased histone lactylation in cardiomyocytes, which fostered ferroptosis by activating the Wnt/β‐catenin pathway. The results of this study may provide a reference for developing novel therapeutic targets for heart diseases. However, this study lacks in vivo experiments to verify the relationship among histone lactylation, the Wnt/β‐catenin pathway, and ferroptosis in heart diseases, which calls for further research. Additionally, our study found that β‐catenin lactylation might inhibit the expression of antioxidant genes such as GPX4 and SLC7A11, reduce GSH levels, and make cells sensitive to lipid peroxidation to promote ferroptosis. Secondly, based on previous studies, we speculated that it might also be attributed to the facts that lactate modification enhances the binding of β‐catenin to iron metabolism genes (such as transferrin receptor TFRC and ferritin light chain FTL) promoters, promotes iron uptake and storage, leading to intracellular free iron accumulation (a key trigger for ferroptosis) [39]. Moreover, it might also be due to the synergistic upregulation of lipoxygenase or inhibition of GPX4 by lactylation and HIF‐1α under hypoxic conditions, which might exacerbate lipid ROS accumulation [40]. This represents a future direction of our further investigation.

## Conflicts of Interest

The authors declare no conflicts of interest.

## Data Availability

The data that support the findings of this study are available from the corresponding author upon reasonable request.
